# Hepatic sEH drives stress-induced neuroinflammation via IL-6 and liver-brain axis

**DOI:** 10.3389/fimmu.2026.1895669

**Published:** 2026-07-24

**Authors:** Shuoqi Yang, Yan Song, Shangge Zhang, Chenyu Liu, Jinghui Cui, Dongmei Wang, Xiaofei Tian, Bin Cong

**Affiliations:** 1 Department of Forensic Medicine, Hebei North University, Zhangjiakou, China; 2 Hebei Key Laboratory of Forensic Medicine, Collaborative Innovation Center of Forensic Medical Molecular Identification, Department of Forensic Medicine, Hebei Medical University, Shijiazhuang, China

**Keywords:** liver–brain axis, microglia, neuronal injury, restraint stress, sEH

## Abstract

**Introduction:**

Restraint stress is known to induce damage to vital organs. Previous work indicates that acute restraint stress (ARS) causes significant hepatic injury in mice. Interestingly, while the liver gradually recovered following removal of the stressor, stress-induced behavioral abnormalities persisted for up to 7 days after stress cessation. Soluble epoxide hydrolase (sEH) plays a key regulatory role in neuroinflammation and mood disorders. However, whether and how hepatic sEH upregulation contributes to ARS-induced neurological damage remains unknown.

**Methods:**

In a mouse model of ARS, behavioral tests were used to assess neurological impairment. Hepatic function was evaluated by blood flow measurement, metabolomics, and serum ALT/AST levels. Hepatic sEH expression was examined by IHC and western blot, and EETs and IL‑6 were quantified with biochemical kits. Microglial activation and neuronal injury in the hypothalamus were assessed by IHC, and IL‑6 expression in hypothalamic neurons was visualized by immunofluorescence double staining.

**Results:**

Restraint stress-induced behavioral abnormalities in mice. These abnormalities persisted for at least 7 days and were accompanied by reduced hepatic blood flow, liver injury, and perturbation of arachidonic acid metabolism. Stress markedly upregulated hepatic sEH expression, decreased epoxyeicosatrienoic acids (EETs), and increased interleukin-6 (IL-6). In the hypothalamus, sEH expression was elevated, microglia exhibited M1-type activation (CD86+), and neuronal loss occurred, while IL-6 levels within hypothalamic neurons was increased. Treatment with the sEH inhibitor TPPU significantly ameliorated stress-induced behavioral abnormalities, liver injury, hypothalamic neuroinflammation, and the abnormal intraneuronal increase of IL-6.

**Discussion:**

These findings reveal that ARS elicits behavioral abnormalities via the liver–brain axis mediated by hepatic sEH upregulation, which reduces EETs and promotes IL-6 release, leading to hypothalamic microglial activation and subsequent neuronal injury. These results identify hepatic sEH as a potential therapeutic target and highlight the liver–brain axis as a critical mediator in ARS-induced behavioral abnormalities and neurological damage.

## 1 Introduction

In forensic practice, cases involving the evaluation of mental disorders resulting from positional restraint, intimidation, or other stressful events are commonly observed (1). Acute restraint stress (ARS) has been shown to enhance the secretion of pro-inflammatory cytokines within the cerebral cortex, thereby precipitating cerebral dysfunction and culminating in neuronal injury (2). Stressful events trigger a neuroendocrine cascade in which the hypothalamic-pituitary-adrenal (HPA) axis becomes activated. This activation drives the systemic release of glucocorticoids and catecholamines (3). HPA axis hyperactivity is frequently associated with depression (4). Stress is also a commonly used model in depression-related research (5–7). Current research on stress has largely focused on the central nervous system. However, stress elicits systemic effects, underscoring the importance of investigating its impact on peripheral vital organs. Previous studies have demonstrated that intense or prolonged stress can induce systemic damage, thereby impairing normal psychological and physiological functions, and leading to pathological injury in vital organs such as the central nervous system (8), heart (9), liver (10), and kidneys (11).

Inflammation is closely associated with psychiatric disorders such as depression and schizophrenia (12, 13), and Soluble epoxide hydrolase (sEH) plays a role in the pathogenesis of inflammation-related diseases (14). The expression and enzymatic activity of sEH vary across different tissues, with the liver exhibiting the highest sEH expression and the strongest enzymatic activity (15). sEH possesses bifunctional enzyme characteristics, exhibiting both epoxyeicosatrienoic acids (EETs) hydrolase activity and lipid phosphatase activity (16). Through these dual pathways, sEH may exacerbate stress-induced behavioral abnormalities: on one hand, the reduction of EETs leads to neuroinflammation and impaired synaptic plasticity (17). On the other hand, dysregulation of lipid phosphate metabolism may affect lipid signal transmission along the liver-brain axis (18). sEH serves as a key metabolic regulatory hub in stress-induced depression (19), hydrolyzing neuroprotective EETs and participating in the regulation of stress responses. Studies have confirmed that interleukin-6 (IL-6) serves as a key proinflammatory node downstream of this regulatory pathway, with its expression closely correlated with sEH activity and EETs levels (20, 21). TPPU is the most commonly used sEH inhibitor (22), characterized by a longer half-life and higher efficacy. The protective effects of TPPU have been validated in various disease models (23–25). Its core mechanism involves inhibition of sEH activity to maintain endogenous EETs levels, thereby exerting significant anti-inflammatory and anti-injury effects. Moreover, studies have confirmed that TPPU effectively alleviates sepsis-associated acute liver injury by precisely modulating the immune microenvironment (26), indicating its targeted potential in hepatic protection. Previous studies have demonstrated that chronic stress selectively upregulates hepatic sEH expression (27). However, whether acute stress-induced hepatic dysfunction (28) is accompanied by elevated sEH expression, and the underlying mechanism of sEH in stress-induced neurological injury, remain to be further elucidated.

Studies have revealed that sEH plays a key regulatory role in neuroinflammation and mood disorders (29). Upregulation of hepatic sEH expression induces significant pathological changes in hepatocytes, while specific inhibition of hepatic sEH alleviates neurological injury symptoms (30). Microglia, as the primary immune effector cells in the central nervous system (31), regulate neuroinflammation through M1/M2 phenotypic polarization (32) and release proinflammatory cytokines and reactive oxygen species (33). Microglia serve as a core hub mediating inflammation-associated neurological damage, and their functional dysregulation directly exacerbates neuronal injury. ARS has been shown to induce significant hepatic dysfunction and pathological changes in the liver (28). Preliminary experiments confirmed that 24 hours of restraint stress significantly upregulated hepatic sEH expression and activated microglia in the hypothalamus.

Therefore, we hypothesize that stress-induced elevated expression of hepatic sEH disrupts EET-mediated neuroprotective signaling and promotes neuroinflammation via IL-6, which in turn, via the liver–brain axis, triggers activation of hypothalamic microglia and neuronal damage, resulting in behavioral abnormalities in mice. To test our hypothesis, we established an ARS model and dynamically observed changes in hepatic sEH expression, EETs and IL-6 levels at various time points after stress exposure, as well as hypothalamic microglial activation status and neuronal damage. Additionally, TPPU intervention was employed to explore the regulatory mechanism of sEH in stress-induced depression via the liver-brain axis.

## 2 Materials and methods

### 2.1 Experimental animals and grouping

The estrous cycle in female mice causes significant fluctuations in the basal activity and stress responsiveness of the hypothalamic-pituitary-adrenal (HPA) axis, which in turn affects baseline glucocorticoid levels and post-stress response intensity, thereby introducing uncontrollable biological variability. To avoid this confounding factor and to ensure the stability and reproducibility of the experimental results, only male mice were used in this study. A total of 70 male specific- pathogen- free (SPF) C57BL/6 mice (8 weeks old, weighing 25 ± 2g) were obtained from Beijing Spefu Biotechnology Co., Ltd. The mice were housed in cages under standard environmental parameters: 12h light/dark cycle, 23°C and 30-70% humidity. Animals received standard pelleted rodent chow (Test Report No.A1F919081A1S42F5723a). Chow and water were provided ad libitum. The research complied with the Institutional Animal Care and Use Committee guidelines and was approved by the Ethics Committee of Academic Committee of Hebei North University (Approval Number: HBNU20260122029). Following acclimatization, the mice were randomly divided into seven groups (n = 10 per group) using a random number generator: control, ARS for 24 hours, and recovery for 3 days, 7 days, or 14 days following stress cessation, as well as a recovery 3 days + TPPU intervention group and a recovery 3 days + ethanol.

### 2.2 Major reagents

TPPU (Cayman Chemical, USA, Cat#11120), Anti-EPXH2 Antibody (Affinity Biosciences, USA, Cat#DF6676; RRID: AB_-_2838638), Anti-Iba1 Antibody (Hangzhou Huaan Biotechnology Co., Ltd., China, Cat#ET1705-78), Anti-CD86 Antibody (Hangzhou Huaan Biotechnology Co., Ltd., China, Cat#ET1606-50), Anti-NeuN Antibody (ABWAYS, China, Cat#CY5515), Anti-IL-6 Antibody (Cohesion Biosciences, United Kingdom, Cat#CQA3710), Nissl staining solution (Beijing Leagene Biotechnology Co., Ltd. Cat#DK0022), Mouse EETs Elisa kit (Animalunion Biotechnology Co., Ltd., Shanghai, China, Cat#LV31290), Mouse IL-6 Elisa kit (Shanghai Jianglai Biotechnology Co., Ltd., China, Cat#JL20268), BCA protein assay kit (Report, China, Cat#RW0201), horseradish peroxidase-conjugated goat anti-rabbit IgG (H+L) (BiYunTian, Shanghai, China, Cat#S1002-100), SV Ultra-sensitive Two-step IHC Kit.(BOSTER Biological Technology Co., Ltd,. USA, Cat#SV0004), DAB (3,3’-diaminobenzidine) Substrate Kit (BOSTER Biological Technology Co., Ltd,. USA, Cat#AR1027-1).

### 2.3 ARS model induction and intervention with the sEH inhibitor TPPU

Following 7 days of acclimatization, mice were subjected to 24 h of restraint stress using transparent acrylic tubes measuring 110 mm in length and 25 mm in inner diameter. These tubes were equipped with adjustable end valves that could be slid to match each animal’s body length, thereby preventing excessive compression of the thorax or abdomen. For ventilation, each valve had circumferential holes approximately 5 mm in diameter, ensuring an uninterrupted supply of fresh air. Visual checks were performed every 2 h, during which no signs of respiratory distress or abnormal behavior were observed. Subsequently, the mice were released from the tubes and allowed to recover under normal feeding conditions. A stock solution of TPPU was prepared by dissolving 5 mg of the crystalline solid in 1 mL of ethanol, and then diluted 10-fold with 9 mL of saline to yield a final concentration of 0.5 mg/mL. Given the short distribution half-life and long elimination half-life of TPPU in mice, it was injected intraperitoneally 30 min before restraint to achieve sEH inhibition prior to stress onset. Mice in the TPPU intervention group received an intraperitoneal injection of TPPU (1.5 mg/kg) 30 min before restraint stress, followed by a second injection immediately after the 24-h stress cessation, and then once daily at the same time for the subsequent 3 days of recovery (totaling five injections). The 3 days+ethanol group received an equivalent volume of 10% ethanol via the same injection schedule.

### 2.4 Open field test

Five mice from each group were randomly selected for the open field test. The open field apparatus (40 cm × 40 cm × 40 cm) was placed in the testing chamber, with the central and peripheral zones predefined. Locomotor activity of the mice was recorded by a high-definition video tracking system mounted above the apparatus. The room was kept quiet, with constant lighting and temperature. Prior to the test, mice were placed in the experimental room for 1 h to acclimate to the environment. At the beginning of the test, each mouse was gently placed into the open field arena. Spontaneous locomotor activity was recorded and analyzed for 6 min, with the entire movement trajectory captured throughout the session. The parameters measured included total distance traveled and time spent in the center. The apparatus was thoroughly cleaned with 75% ethanol between trials to eliminate olfactory cues.

### 2.5 Tail suspension test

For the tail suspension test, the remaining five mice per group were suspended by the tail 50 cm above the chamber floor, with adhesive tape placed less than 1 cm from the tail tip. Behavior was video-recorded for 6 min, and immobility time was recorded and analyzed. Increased immobility time indicates elevated helplessness.

### 2.6 Tissue specimen collection

Mice were anesthetized with 1% sodium pentobarbital (50 mg/kg, i.p.). Approximately 0.8 mL of blood was collected via the retro-orbital sinus and left to stand at room temperature for 30 min to clot. Serum was then separated by centrifugation at 12,000 rpm (approximately 13,206 ×g) for 20 min at 4 °C. Subsequently, the whole brain was rapidly removed on ice and immersion-fixed together with one intact liver lobe in 4% paraformaldehyde for 24 hours. The fixed brain was then trimmed coronally, guided by The Mouse Brain in Stereotaxic Coordinates (Paxinos and Franklin, 2nd edition), centering the block at 2.46 mm anterior to the interaural line and 1.34 mm posterior to bregma to produce a tissue slab encompassing the hypothalamus. This brain slab and the liver tissue were then dehydrated, cleared, and embedded in paraffin. Coronal brain sections and liver sections were prepared for subsequent morphological analysis. All remaining liver tissue and the collected serum samples were stored at −80 °C until further analysis.

### 2.7 Liver blood flow determination

LBF was measured at baseline using a laser Doppler perfusion imager (PeriCam PSI NR, PSIN‐01104, Sweden) equipped with a computer. The scanner head was positioned 15 cm from the liver. The beam illuminated the tissues to a depth of 0.5 mm. The magnitude of liver blood flow was represented by different colors, with blue and red denoting low and high blood flow, respectively. A rectangle region (1.5 × 1.5 cm, the center of the left lateral lobe) including the main branch of the microcirculatory network was outlined on each image and used to calculate the area‐averaged flux. Data were presented as mean flux from the measured region in perfusion units.

### 2.8 Metabonomics

Mice were anesthetized and sacrificed, and liver tissues were immediately collected and frozen in liquid nitrogen. Metabolomic analysis was performed by Shanghai Applied Protein Technology Co. Ltd. All differentially expressed metabolites were subjected to heatmap analysis.

### 2.9 H&E staining

Paraffin-embedded liver and sections were baked at 60 °C for 30 min, deparaffinized, and rehydrated. Sections were stained with hematoxylin for 5 min, differentiated in 1% acid alcohol for 30 s, and blued in tap water. Subsequently, sections were stained with 1% eosin for 3 min, dehydrated through graded ethanol, cleared in xylene, and mounted with neutral balsam. Stained sections were examined and photographed under a microscope.

### 2.10 Biochemical analysis of EETs

A blank well received 50 μL of standard/sample diluent, while the remaining wells were filled with 50 μL of either standards or samples. The plate was sealed with adhesive film, mixed gently, and incubated at 37 °C for 50 min. After incubation, the plate was washed three times with 1× wash buffer (300 μL per well), each wash accompanied by shaking for 1 min, and then blotted dry on absorbent paper. The blank well received 100 μL of biotinylated antibody diluent, while the other wells received 100 μL of 1× biotinylated antibody working solution. The plate was sealed, mixed, and incubated at 37 °C for 50 min. Following another washing step as described above, the plate was inverted and tapped firmly onto absorbent paper to remove residual liquid. Each well was then filled with at least 300 μL of 1× wash buffer and allowed to soak for 1–2 min. Subsequently, 100 μL of SABC complex working solution was added to each well, the plate was sealed, mixed, and incubated at 37 °C for 30 min. After another washing step, 100 μL of freshly prepared TMB substrate solution was added to each well. The plate was sealed, mixed, and incubated in the dark at 37 °C for 10–20 min, with the development time adjusted according to color intensity. Finally, 50 μL of stop solution was added to each well, mixed gently, and the absorbance was measured at 450 nm using a microplate reader within 30 min.

### 2.11 Biochemical analysis of IL-6

Required strips were removed from the aluminum foil bag after equilibration at room temperature for 20 min, and the remaining strips were sealed in a resealable bag and stored at 4 °C. Standard wells and sample wells were designated. Standard wells received 50 μL of standards at various concentrations, sample wells received 50 μL of test samples, and blank wells received no addition. Except for blank wells, each standard well and sample well received 100 μL of horseradish peroxidase (HRP)-conjugated detection antibody. The plate was sealed with adhesive film and incubated at 37 °C for 60 min in a water bath or incubator. After incubation, the liquid was discarded, and the plate was blotted dry on absorbent paper. Each well was filled with 350 μL of wash buffer, allowed to stand for 1 min, and then the wash buffer was discarded, followed by blotting on absorbent paper. This washing procedure was repeated five times. Subsequently, 50 μL of substrate A and 50 μL of substrate B were added to each well, and the plate was incubated in the dark at 37 °C for 15 min. Finally, 50 μL of stop solution was added to each well, and the optical density (OD) of each well was measured at 450 nm within 15 min. A standard curve was generated, and a linear regression equation was derived to calculate sample concentrations.

### 2.12 Nissl staining

Brain tissue sections were routinely deparaffinized and rehydrated to water. The sections were then placed in Cresyl Violet stain solution and incubated in a 56 °C oven for 1 h. After staining, the sections were transferred to Nissl Differentiation solution for 1–3 min, with differentiation monitored under a microscope until the background became nearly colorless. The sections were then dehydrated in absolute ethanol, cleared in xylene, and mounted with neutral balsam. The number of normal neurons in the hypothalamus was counted using ImageJ software. Five non-overlapping brain sections were analyzed per animal.

### 2.13 Immunohistochemistry

For immunohistochemical analysis, brain sections underwent a gradient dewaxing process followed by antigen retrieval using citrate buffer. Post retrieval, sections were washed with PBS three times and sequentially treated with 3% hydrogen peroxide and 10% goat serum to block nonspecific binding. Sections were then incubated overnight at 4^°^C with anti-sEH (1:300), anti-Iba1 (1:1000), anti-CD86 (1:500). After incubation, sections were allowed to return to room temperature for 30 min and washed three times with PBS for 3 min each. Biotin-labeled goat anti-rabbit IgG secondary antibody working solution was added and incubated at room temperature for 50 min. After PBS washing three times, using DAB to develop color and hematoxylin was used to stain the nucleus. After dehydration and transparency, it is sealed with neutral gum. After drying, the slide is placed in a panoramic scanning image system for full slide scanning. The expression of sEH in hepatic and hypothalamic was evaluated by measuring the integrated optical density. Microglial morphology and coverage were quantified as the percentage of positive area, which includes both the soma and all processes. CD86-positive microglia were quantified by direct cell counting. All quantitative analyses of the hypothalamus were performed using ImageJ software, and the operator was blinded to experimental group allocation. Five non-overlapping brain sections were analyzed per animal.

### 2.14 Western blotting

Liver tissue (0.02 g) was homogenized mechanically in 200 μL of ice-cold 1× RIPA lysis buffer containing protease inhibitors and left on ice for 30 min. Lysates were centrifuged at 12,000 rpm for 20 min at 4 °C, and supernatants were stored at −80 °C. Protein concentrations were determined by BCA assay. Equal amounts of protein (30 μg) were separated by SDS-PAGE using a 5% stacking gel and an 8% resolving gel. Electrophoresis was performed at 80 V through the stacking gel (~1.5 h) and then at 120 V through the resolving gel until the dye front reached the bottom. Proteins were transferred onto PVDF membranes by wet transfer at a constant current of 220 mA for 1 h in an ice-cold transfer buffer. After transfer, membranes were blocked with 5% non-fat milk in TBST for 2 h at room temperature and washed three times with TBST (5 min each). Based on molecular weight markers, the membranes were cut horizontally at approximately 63 kDa and 36 kDa to separate the sEH- and GAPDH-containing regions. The sEH strip was incubated overnight at 4 °C with rabbit anti-sEH (1:20,000). After rewarming to room temperature for 30 min and washing, the membrane was incubated with goat anti-rabbit IgG-HRP (1:8,000) for 2.5 h at 37 °C, followed by three additional washes. The GAPDH strip was processed in parallel with rabbit anti-GAPDH (1:80,000) and goat anti-rabbit IgG-HRP (1:8,000) for 2.5 h at 37 °C as a loading control. Signals were developed with ECL reagent and imaged using an automated chemiluminescence imaging system. Band intensities were quantified with ImageJ and normalized to GAPDH.

### 2.15 Immunofluorescence staining

Sections were routinely deparaffinized and rehydrated. Antigen retrieval was performed using citrate buffer in a microwave. After cooling to room temperature, sections were rinsed with PBS. Endogenous peroxidase activity was blocked by incubation with 3% hydrogen peroxide at room temperature for 20 min, followed by PBS rinsing. Sections were then permeabilized with 0.1% Triton X-100 for 10 min and blocked with 10% goat serum for 30 min at 37°C. Primary antibodies (anti-NeuN antibody at 1:200 dilution and anti-IL-6 antibody at 1:100 dilution) were applied, and sections were incubated overnight at 4 °C. After incubation, sections were allowed to rewarm at room temperature for 30 min and rinsed with PBS. HRP-conjugated goat anti-rabbit IgG secondary antibody was applied and incubated at room temperature for 1 hour. After PBS washing, sections were mounted with anti-fade mounting medium containing DAPI. Staining was examined under a fluorescence microscope. Images were captured at 400 × magnification, and the relative fluorescence intensity of positive cells was quantified using ImageJ software.

### 2.16 Statistical methods

All data were expressed as mean ± SD and analyzed using GraphPad Prism software. One-way analysis of variance (ANOVA) followed by Tukey’s *post hoc* test was used for comparisons among the Control, 24 hours, 3 days, 7 days, and 14 days groups. Two-way ANOVA followed by Tukey’s *post hoc* test was used for comparisons among the Control, 3 days, and 3 days+TPPU groups. A two-tailed P value < 0.05 was considered statistically significant.

## 3 Results

### 3.1 Restraint stress induces depression-like symptoms in mice

**Figure 1:**
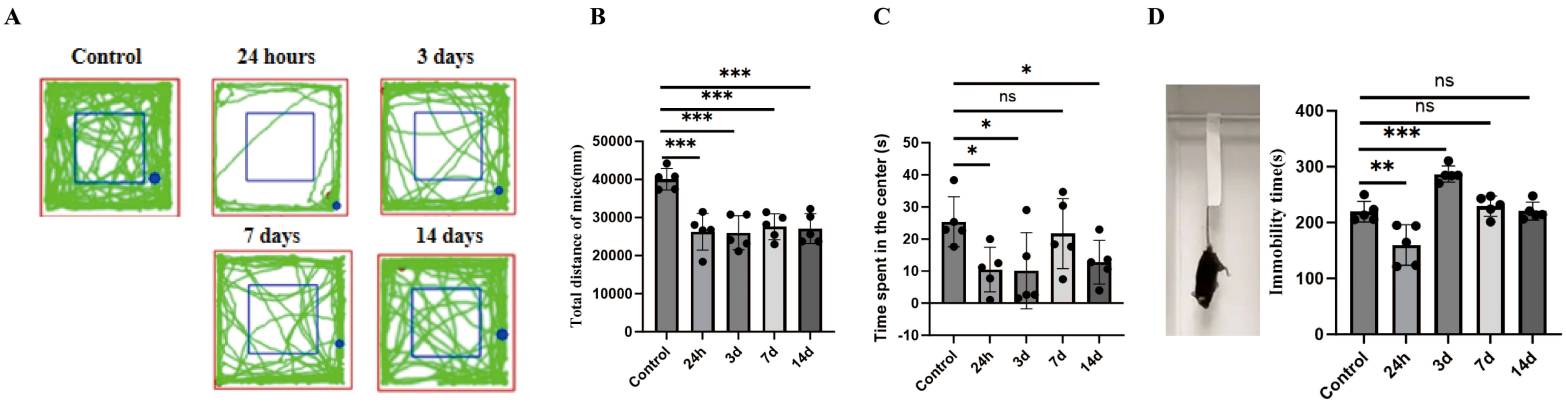
Restraint stress induces depression-like symptoms in mice. **(A)** Locomotor trajectory maps. **(B)** Total distance traveled (n=5 mice/group). **(C)** Time spent in the center (n=5 mice/group). **(D)** Immobility time in the tail suspension test(n=5 mice/group). Data are presented as mean ± SD. Significance was calculated by one-way ANOVA followed by Tukey’s *post hoc* test. *p<0.05; **p<0.01; ***p<0.001 vs. Control group.

To investigate the influence of ARS on emotional behavior in mice, we initially employed the open field test. Stressed mice demonstrated locomotor activity predominantly confined to the peripheral zone of the arena (**Figure 1A**). In comparison to the control group, stressed mice exhibited a significant reduction in both total traveled distance and time spent in the center (*P* < 0.01, **Figures 1B, C**). Additionally, the tail suspension test revealed that stressed mice displayed a significant increase in immobility time (*P* < 0.01, **Figure 1D**), indicating enhanced behavioral despair. These data demonstrate that restraint stress induces behavioral abnormalities in mice, and that these behavioral deficits gradually ameliorate with increasing duration of recovery.

### 3.2 Restraint stress-induced hepatic injury and metabolic disturbances in mice

**Figure 2:**
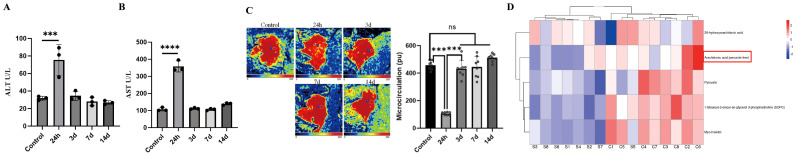
Restraint stress induces hepatic injury and metabolic disturbances in mice. **(A)** Serum alanine aminotransferase (ALT). **(B)** Serum aspartate aminotransferase (AST). **(C)** Hepatic blood flow measured by laser Doppler perfusion imaging. **(D)** Heatmap of hepatic metabolites (S_1-8_: stress group; C_1-8_: control group). Data are presented as mean ± SD. Significance was calculated by one-way ANOVA followed by Tukey’s *post hoc* test. ***p<0.001; ****p<0.0001 vs. Control group.

Serum levels of Alanine Aminotransferase (ALT) and Aspartate Aminotransferase (AST) were measured using biochemical assays. The results showed that ARS significantly increased serum ALT (*P* < 0.001, **Figure 2A**) and AST levels (*P* < 0.001, **Figure 2B**) in mice. Using Doppler perfusion imaging, we found that 24 hours of continuous restraint stress significantly reduced hepatic blood flow in mice (*P* < 0.001, **Figure 2C**). To further elucidate the effects of ARS on hepatic function, we performed metabolomic analysis of liver tissues. The results revealed a distinct clustering separation in the hepatic metabolic profiles between the stress group and the control group. Notably, arachidonic acid levels were significantly decreased in the stress group (**Figure 2D**).

### 3.3 Restraint stress in mice was found to upregulate hepatic sEH expression, leading to a concomitant decrease in EETs, along with increased IL-6 levels in both the liver and serum

**Figure 3:**
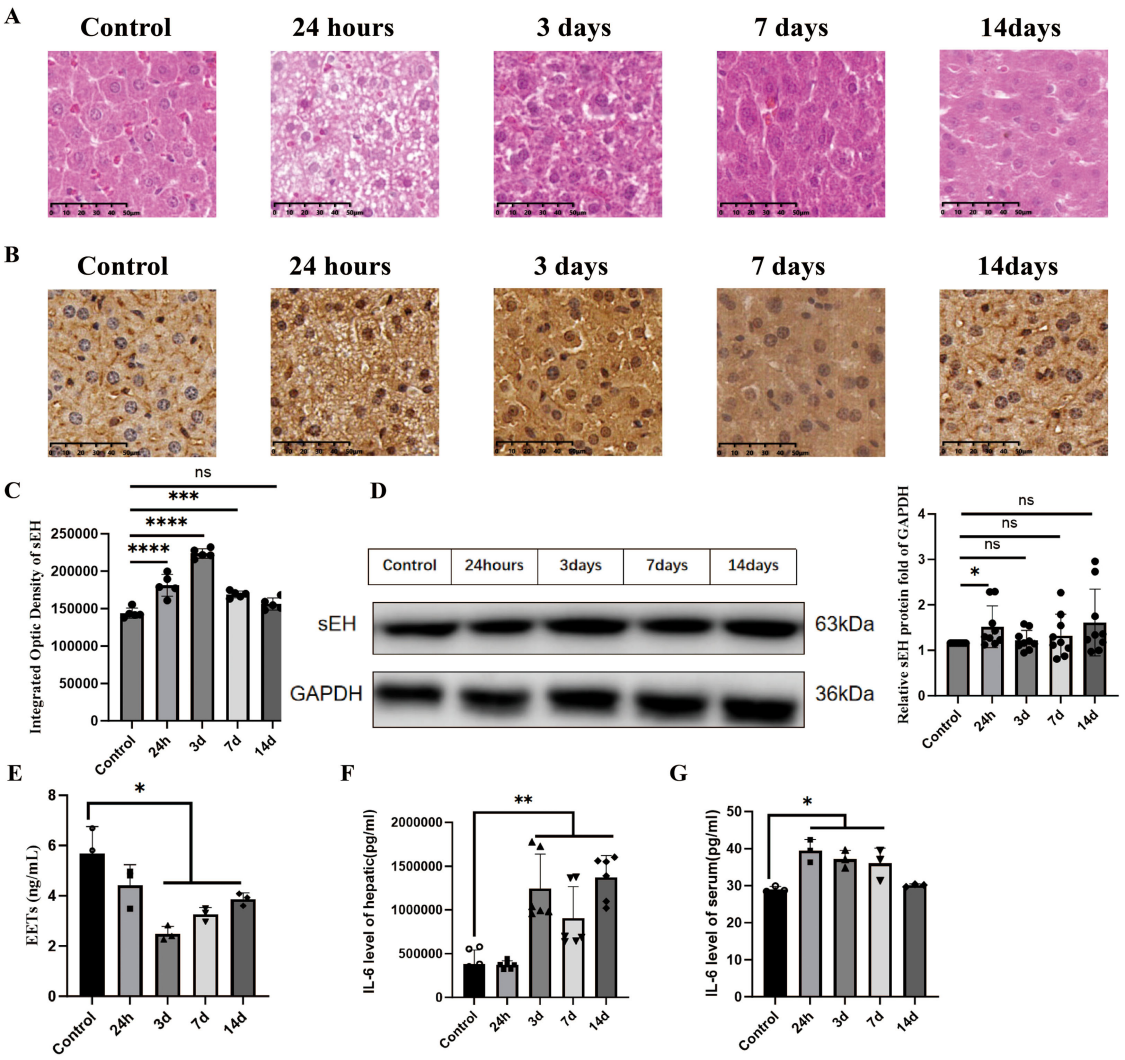
Restraint stress in mice was found to upregulate hepatic soluble epoxide hydrolase (sEH) expression, leading to a concomitant decrease in epoxyeicosatrienoic acids (EETs), along with increased IL-6 levels in both the liver and serum. **(A)** H&E staining of liver sEH(400×, scale bars, 50 μm.). **(B, C)** Immunohistochemistry for hepatic (400×). **(D)** Western blot analysis of sEH expression. **(E)** Hepatic EETs levels. **(F)** Hepatic IL-6 levels. **(G)** IL-6 levels of serum. Quantification of Immunohistochemistry (n = 5 brain sections/group). Scale bars, 50 μm. Data are presented as mean ± SD. Significance was calculated by one-way ANOVA followed by Tukey’s *post hoc* test. *p<0.05; **p<0.01; ***p<0.001; ****p<0.0001 vs. Control group.

Hematoxylin and eosin (HE) staining revealed that ARS resulted in marked hydropic degeneration of hepatocytes and disruption of nuclear membrane integrity (**Figure 3A**). Consistent with these pathological findings, hepatic sEH expression was significantly increased following stress exposure (*P* < 0.05, **Figures 3B–D**). Importantly, both the stress-induced hepatic injury and the upregulation of sEH exhibited a gradual recovery trend upon cessation of the restraint stimulus. Quantitative analysis using commercial kits demonstrated that ARS led to a significant reduction in hepatic epoxyeicosatrienoic acids (EETs) and a concomitant increase in interleukin-6 (IL-6) levels within liver tissue (*P* < 0.05, **Figures 3E, F**). Moreover, this proinflammatory response extended to the periphery, as evidenced by a significant elevation of serum IL-6 levels in stressed mice (*P* < 0.05, **Figure 3G**).

### 3.4 Restraint stress induces a reduction of hypothalamic neurons and pronounced microglial activation accompanied by upregulation of sEH in mice

**Figure 4:**
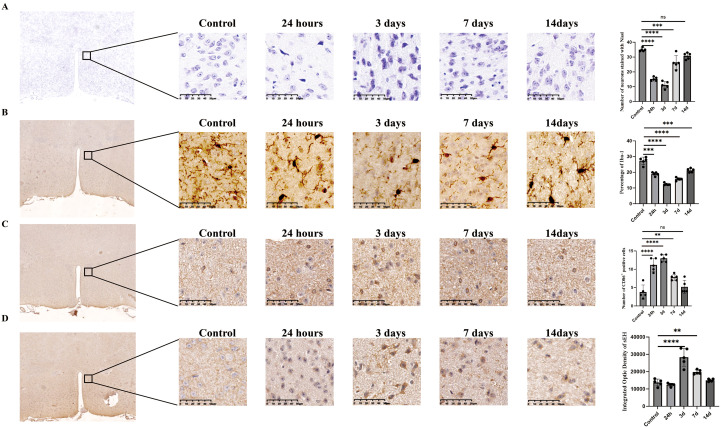
Restraint stress induces a reduction of hypothalamic neurons and pronounced microglial activation accompanied by upregulation of sEH in mice. **(A)** Nissl staining of the hypothalamus (400×). **(B)** Immunohistochemical for the microglial marker Iba-1 in the hypothalamus (400×). **(C)** Immunohistochemical for microglial CD86 in the hypothalamus (400×). **(D)** Immunohistochemical for sEH in the hypothalamus (400×). Quantification of Nissl staining and Immunohistochemistry (n = 5 brain sections/group). Scale bars, 50 μm. Data are presented as mean ± SD. Significance was calculated by one-way ANOVA followed by Tukey’s *post hoc* test. **p<0.01; ***p<0.001; ****p<0.0001 vs. Control group.

Evaluation of hypothalamic neuronal integrity by Nissl staining demonstrated that ARS resulted in a significant reduction in neuron numbers. Notably, this neuronal loss exhibited a time-dependent recovery trend following the cessation of stress exposure (*P* < 0.01, **Figure 4A**). Immunohistochemical examination of hypothalamic microglia revealed that ARS induced robust microglial activation. Activated microglia exhibited characteristic morphological changes, including cell body enlargement, significant shortening of average process length, and marked reductions in both branch points and terminal branches (*P* < 0.05, **Figure 4B**). Furthermore, the expression of CD86, a canonical marker of M1-type microglial polarization, was significantly elevated following stress exposure (*P* < 0.01, **Figure 4C**). Interestingly, CD86 expression reached its maximum in the group allowed to recover for 3 days after stress cessation. Immunohistochemical analysis of hypothalamic sEH expression showed no significant change at 24 h after acute stress compared with the control group, but a significant increase was observed in the 3 days group (P < 0.05, **Figure 4D**).

### 3.5 TPPU intervention ameliorated stress-induced behavioral abnormalities and hepatic injury in mice

**Figure 5:**
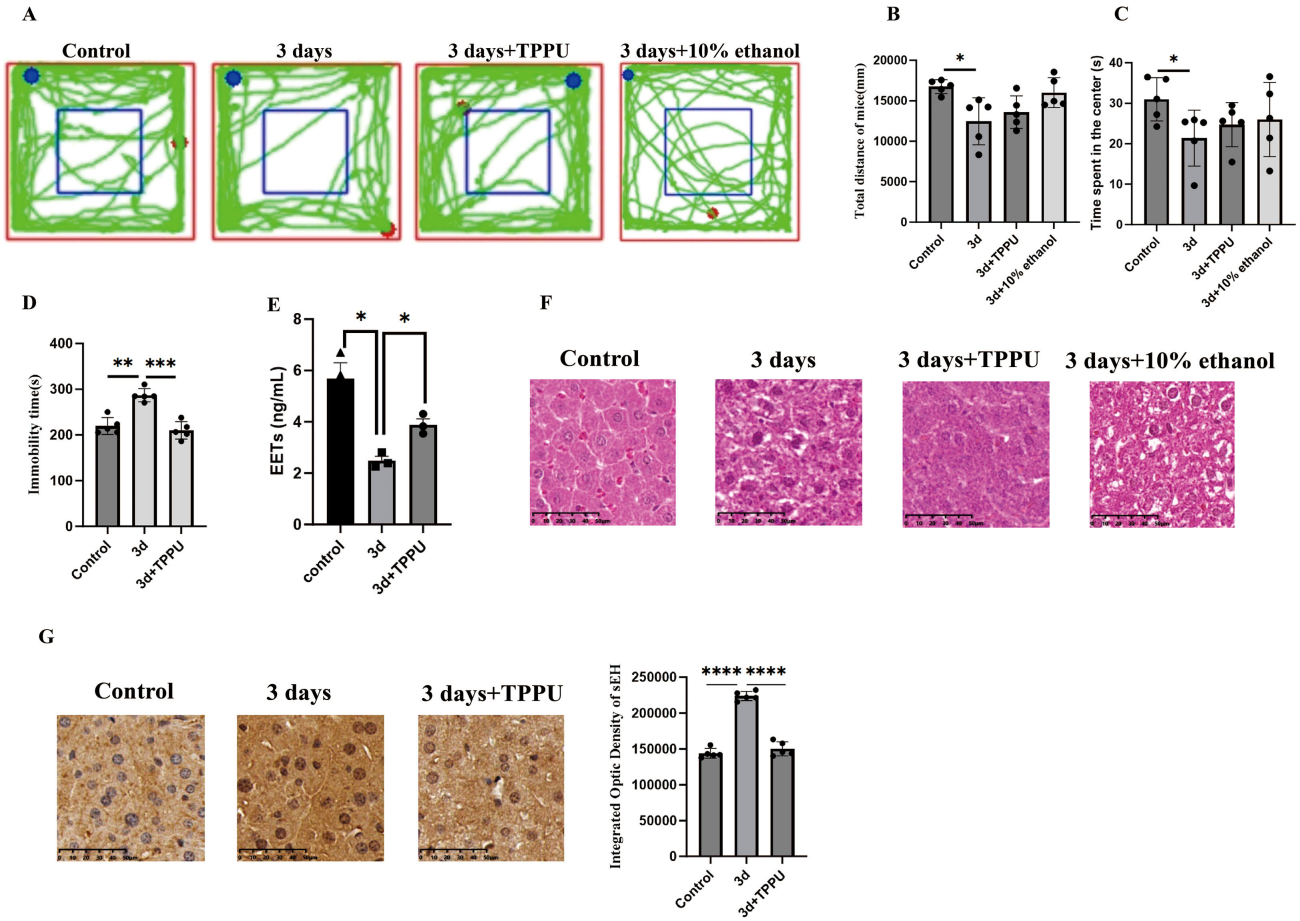
sEH inhibitor TPPU ameliorates stress-induced behavioral abnormalities and hepatic sEH upregulation in mice. **(A)** Locomotor trajectory maps. **(B)** Total distance traveled (n=5 mice/group). **(C)** Time spent in the center (n=5 mice/group). **(D)** Immobility time in the tail suspension test (n=5 mice/group). **(E)** Hepatic EETs levels. **(F)** H&E staining of liver (400×, scale bars, 50 μm.). **(G)** Immunohistochemistry for hepatic sEH (400×). Quantification of Immunohistochemistry (n = 5 brain sections/group). Scale bars, 50 μm. Data are presented as mean ± SD. Significance was calculated by two-way ANOVA followed by Tukey’s *post hoc* test among the Control, 3 days, and 3 days+TPPU groups. *p<0.05; **p<0.01; ***p<0.001; ****p<0.0001.

The above experimental results indicated that pathological changes in the hypothalamus were most pronounced in the 3 days group following removal of restraining factors, leading us to select the 3 days group for further intervention with the sEH inhibitor TPPU. Behavioral testing showed increased movement trajectories in the central area in TPPU-treated mice (**Figure 5A**). There were no significant differences in total distance traveled, time spent in the center, or immobility time compared with the control group, indicating a recovery to baseline levels (P<0.05, **Figures 5B–D**). Concurrently, hepatic EETs levels in the 3 days+TPPU group were significantly higher than in the 3 days group (P<0.05, **Figure 5E**), and hepatocellular edema was partially ameliorated (**Figure 5F**). Immunohistochemical assessment of sEH expression revealed that hepatic sEH expression in the 3 days+TPPU group was reduced compared with the 3 days group (P<0.0001, **Figure 5G**) and showed no statistically significant difference from the control group.

### 3.6 TPPU intervention attenuated stress-induced activation of hypothalamic microglia and alterations in sEH expression in mice

**Figure 6:**
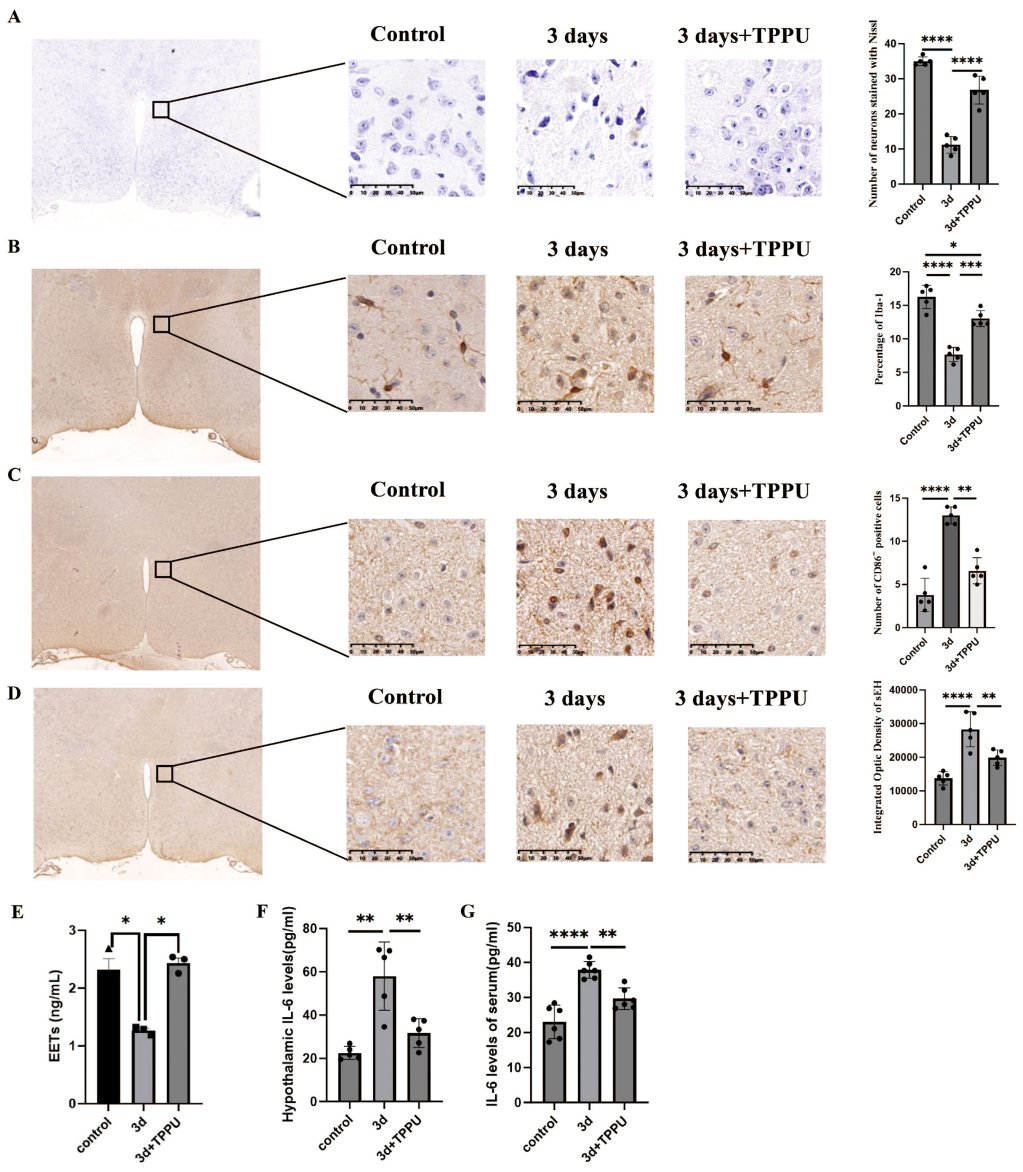
TPPU, an sEH inhibitor, reduces the activated microglia in the hypothalamus of stress-exposed mice while sEH expression remains unchanged. **(A)** Nissl staining of the hypothalamus (400×). **(B)** Immunohistochemical for the microglial marker Iba-1 in the hypothalamus (400×). **(C)** Immunohistochemical for microglial CD86 in the hypothalamus (400×). **(D)** Immunohistochemical for sEH in the hypothalamus (400×). **(E)** Hypothalamic EETs levels. **(F)** Hypothalamic IL-6 levels. **(G)** IL-6 levels of serum. Quantification of Nissl staining and Immunohistochemistry (n = 5 brain sections/group). Scale bars, 50 μm. Data are presented as mean ± SD. Significance was calculated by two-way ANOVA followed by Tukey’s *post hoc* test among the Control, 3 days, and 3 days+TPPU groups. *p<0.05, **p<0.01, ***p<0.001, ****p<0.0001.

Further Nissl staining analysis revealed a significantly higher neuronal number in the 3 days+TPPU group compared with the 3 days group (P<0.0001, **Figure 6A**). However, Immunohistochemical analysis of the Iba-1-positive microglial area was significantly increased (P<0.001, **Figure 6B**), while the expression of the M1-type activation marker CD86 was markedly reduced (P<0.01, **Figure 6C**), with both parameters restored to levels approximating the control group. Immunohistochemical analysis of hypothalamic sEH expression revealed a decrease in the 3 days group compared with the 3 days+TPPU group (P < 0.01, **Figure 6D**). Further examination of hypothalamic EETs levels showed that they were significantly lower in the 3 days group than in the control group, but were restored to control levels after TPPU treatment (P<0.05, **Figure 6E**). And TPPU treatment significantly suppressed IL-6 expression in the hypothalamus (P < 0.01, **Figure 6F**) and reduced serum IL-6 levels (P < 0.01, **Figure 6G**).

### 3.7 TPPU intervention attenuated stress-induced upregulation of IL-6 levels in hypothalamic neurons of mice

**Figure 7:**
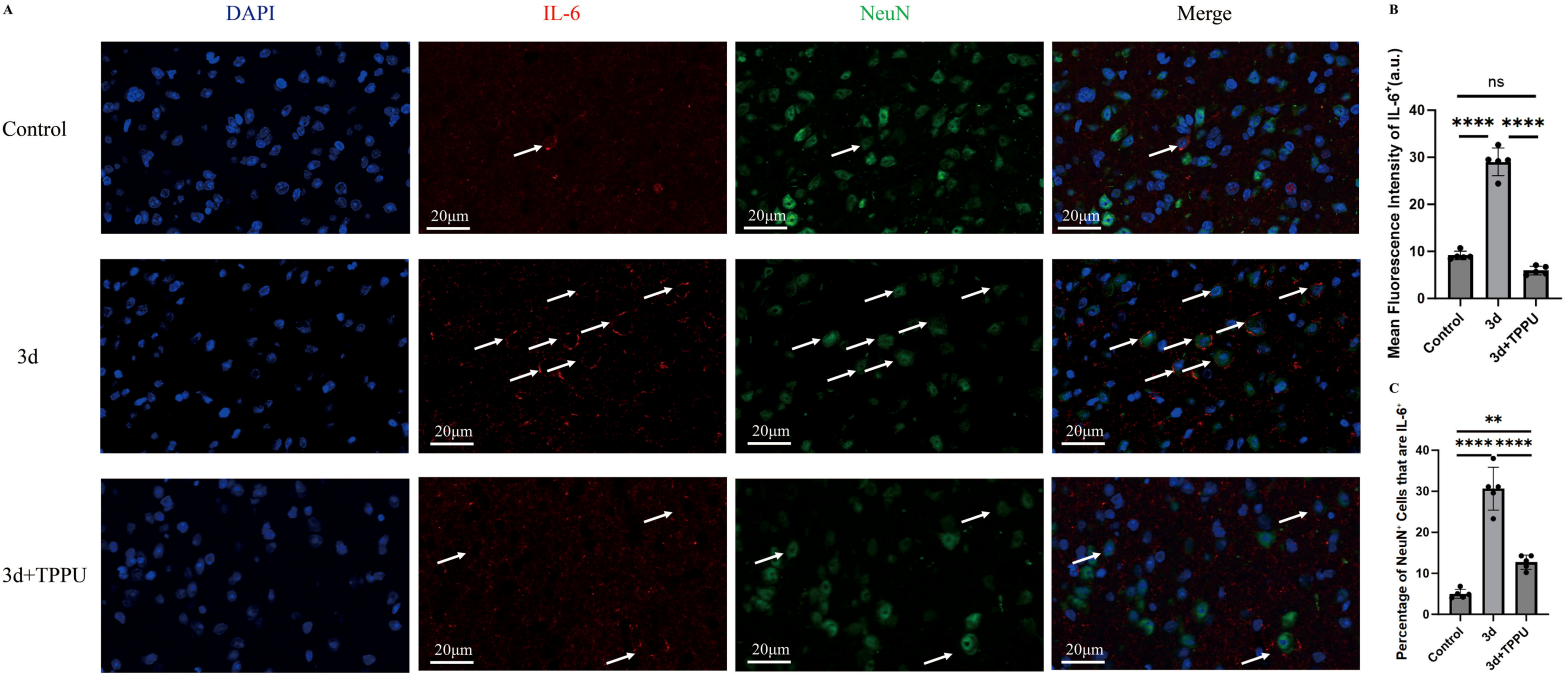
After intervention with the sEH inhibitor TPPU, IL-6 levels in hypothalamic neurons of stress-exposed mice was reduced. **(A)** Immunofluorescence double staining for IL-6 (red) and NeuN (green) in hypothalamus (400×). **(B)** Mean fluorescence intensity of IL-6 in hypothalamic neurons. **(C)** Number of IL-6/NeuN co-positive cells in hypothalamus. Quantification of Immunofluorescence staining (n = 5 brain sections/group). Scale bars, 20 μm. Data are presented as mean ± SD. Significance was calculated by two-way ANOVA followed by Tukey’s *post hoc* test among the Control, 3 days, and 3 days+TPPU groups. **p<0.01, ****p<0.0001.

The results have confirmed that hepatic sEH is highly expressed while EETs are reduced and IL-6 is elevated, with serum IL-6 also markedly higher than in controls. We further assessed IL-6 content in hypothalamic neurons by immunofluorescent double-labeling, and found that restraint stress induced a marked increase of IL-6 in hypothalamic neuronal cells of mice (**Figure 7A**). Specifically, the mean fluorescence intensity of IL-6 in hypothalamic neurons was significantly increased in the 3 days group, whereas it was significantly reduced in the 3 days + TPPU intervention group compared with the 3 days group (P<0.0001, **Figure 7B**), and did not differ significantly from the Control group (P>0.05, **Figure 7B**). The number of IL-6/NeuN co-labeled cells in the hypothalamus was also significantly reduced in the 3 days + TPPU group compared with the 3 days group (P<0.0001, **Figure 7C**).

## 4 Discussion

In recent years, the mechanisms underlying stress-induced liver and brain injury have increasingly been highlighted as a key area of research (34, 35). This investigation of brain injury demonstrates that specifically inhibiting sEH expression in the liver effectively alleviates central nervous system lesions (30). Our previous study demonstrated that restraint stress causes marked hepatic dysfunction and pathological alterations in mice (28). Additionally, studies have shown that acute restraint stress (ARS) can lead to psychiatric abnormalities in mice that persist for up to 35 days (36). Based on our preliminary findings that ARS induces elevated expression of sEH in the mouse liver, the study investigates the hypothesis that elevated hepatic sEH expression resulting from ARS mediates stress-induced neurological damage via the liver–brain axis.

Using the open field test and tail suspension test, we first confirmed that ARS induces behavioral abnormalities in mice, a finding consistent with previous reports that stress triggers emotional behavioral abnormalities (37). Further examination revealed that restraint stress reduced hepatic blood flow per unit area, elevated serum ALT and AST levels, and induced hepatocyte edema along with nuclear membrane disruption, indicating impaired liver function. It is worth noting that the time course of recovery from stress differs significantly between peripheral organs and the central nervous system. Hepatic injury began to recover on day 3 after stress and largely normalized by day 7. In contrast, stress-induced behavioral abnormalities persisted for at least 7 days, consistent with previous reports that acute restraint stress-induced behavioral abnormalities can last up to 35 days. It has been shown that chronic liver disease can promote neuroinflammation through sustained systemic inflammatory signaling (38). Furthermore, neuroinflammation can also occur independently of peripheral inflammation in certain conditions (39). Consistent with this notion, our findings suggest that persistent behavioral abnormalities after stress cessation does not necessarily depend on ongoing hepatic injury, but may instead be triggered by inflammatory signals originating from the liver during the early phase of acute stress.

To gain further insight into the hepatic consequences of ARS, we conducted untargeted metabolomic profiling of liver tissue. The analysis demonstrated a significant reduction in arachidonic acid levels in the restraint stress group. Arachidonic acid is metabolized by cytochrome P450 enzymes to form EETs, which are known substrates of sEH, suggesting a disruption in this metabolic pathway under stress conditions (26). As a key enzyme in this pathway, hepatic sEH expression was significantly upregulated at 24 h after acute stress. This is consistent with the work of Qin et al. (27), who first showed that chronic stress selectively upregulates hepatic sEH, and that liver-specific elevated expression is sufficient to induce depressive-like behavior. Here, we extend this concept to acute stress, showing that hepatic sEH upregulation is a rapid stress response occurring within the first 24 h. sEH is a bifunctional enzyme with epoxide hydrolase and lipid phosphatase activities (40). By converting EETs and other epoxy fatty acids into their corresponding inactive diols, sEH depletes these anti-inflammatory mediators, thereby directly impairing the local anti-inflammatory capacity of the liver and leading to pathological changes such as hepatocyte edema and nuclear membrane damage. Consistent with the EETs assay results, we confirmed that the functional elevation of hepatic sEH diminishes the abundance of anti-inflammatory EETs. IL-6 is a multifunctional cytokine that plays a central role in inflammatory responses through activation of the JAK/STAT3, MAPK, and PI3K/AKT signaling pathways (41). Subsequent examination of IL-6 in liver tissue and serum demonstrated that restraint stress elevated hepatic IL-6 levels, and serum IL-6 levels were also sustained at a high level. Collectively, this study demonstrates that rapid hepatic sEH upregulation under acute stress drives excessive IL−6 production by depleting anti−inflammatory EETs.

The hypothalamus, acting as a pivotal hub for neuroendocrine regulation, represents the core responsive site in the central nervous system under stress conditions (42). The paraventricular nucleus (PVN) of the hypothalamus, via corticotropin-releasing hormone release, activates the HPA axis cascade, integrating central and peripheral signals to orchestrate the neuroendocrine response to stress (43). Nissl staining revealed that the number of hypothalamic neurons began to decline as early as 24 h after stress, reaching a nadir on day 3. Microglia function as the primary immune effector cells within the central nervous system (44). Immunohistochemistry for Iba-1 and CD86 revealed that hypothalamic microglial M1 polarization occurred as early as 24 h after stress and peaked on day 3, characterized by enlarged cell bodies and shortened processes, consistent with a morphological shift from a ramified resting state to an amoeboid phenotype. This morphological transformation involved pronounced process retraction, which markedly reduced the area occupied by individual cells in tissue sections, thereby accounting for the significant decrease in Iba-1-positive area fraction. Consistent with recently reported findings, acute stress has been reported to activate microglia in the brain, particularly in the hypothalamus, a core region for stress responses (45). Upon M1 polarization, microglia produce IL-1β, IL-6, and TNF-α, among other cytokines. The combined action of these factors drives neuronal death, disrupts the blood–brain barrier, and propagates cerebral tissue damage (46). Our findings demonstrated that restraint stress led to persistently high serum IL-6 levels, with IL-6 levels also detected in hypothalamic neurons. Quantitative analysis showed that the number of NeuN/IL-6 double-positive neurons in the hypothalamus was significantly higher in the ARS group than in controls, along with a significant increase in IL-6 levels. These observations are consistent with previous evidence that peripheral IL-6 can access the brain via a disrupted blood–brain barrier (47, 48). Furthermore, peripheral inflammatory signals can act directly on the hypothalamus through a faster neural reflex circuit. Cytokines like IL-6 released in the periphery can activate vagal afferents, with signals being relayed via the nucleus tractus solitarius (NTS) to directly innervate and stimulate the PVN of the hypothalamus (49). Subdiaphragmatic vagotomy abolishes the transmission of peripheral inflammatory signals to the hypothalamus (50), providing direct evidence that the vagus nerve serves as an essential pathway for conveying intra-abdominal peripheral immune signals to the brain. In addition, sympathetic activation can trigger substantial local release of norepinephrine in the hypothalamic PVN. Previous studies have established that norepinephrine is a key neurotransmitter regulating microglial activation. Stress engages the sympathetic nervous system to trigger norepinephrine release, which, acting through β-adrenergic receptors, directly drives the transition of microglia toward an activated phenotype (51, 52). Therefore, in the early stage of restraint stress, rapid microglial M1 polarization is likely initiated, at least in part, by the vagal-sympathetic reflex, rather than depending exclusively on the passive entry of circulating factors through a damaged blood–brain barrier. Analysis of hypothalamic sEH expression revealed that, unlike the rapid upregulation of hepatic sEH at 24 h, hypothalamic sEH levels did not differ significantly from the control group at 24 h and were significantly elevated only by day 3. This delayed increase in hypothalamic sEH occurred after the onset of microglial activation and neuronal injury, suggesting that it may represent a secondary event that exacerbates microglial activation by hydrolyzing local anti-inflammatory EETs, thereby amplifying or sustaining the inflammatory cascade (17, 53). M1 microglia produce abundant pro-inflammatory cytokines and reactive oxygen species (54), leading directly to neuronal damage and synaptic plasticity deficits in the hypothalamus, which in turn precipitate behavioral abnormalities (55). Collectively, early upregulation of hepatic sEH and the consequent rise in IL-6 contribute to initiating the cascade that leads to sustained hypothalamic injury, whereas the delayed upregulation of hypothalamic sEH exacerbates pre-existing neuroinflammation.

To further validate the regulatory role of hepatic sEH, this study administered the sEH-specific inhibitor TPPU to the group evaluated 3 days after removal of the stressor. TPPU, through inhibition of sEH activity, elevates EETs levels and confers anti-inflammatory and neuroprotective effects (23). TPPU intervention ameliorated stress-induced behavioral abnormalities in mice, with decreased hepatic sEH expression, restored EETs levels, and mitigation of hepatocyte edema. Furthermore, the Iba-1-positive area fraction increased, accompanied by significant downregulation of CD86, a hallmark of M1 microglial activation. Importantly, TPPU intervention markedly abrogated the upregulation of IL-6 within hypothalamic neurons. These observations further support a neuroprotective role for hepatic sEH and validate the proposed liver–brain axis injury mechanism, establishing hepatic sEH as a key upstream regulator of stress-induced neuroinflammation. Early targeting of hepatic sEH may represent a more effective strategy for interrupting the transmission of pro-inflammatory signals from the periphery to the central nervous system, thereby conferring neuroprotection.

Previous studies have largely concentrated on the effects of chronic stress on brain regions such as the hippocampus (56) and the amygdala (57). However, the mechanisms underlying hypothalamic injury induced by acute stress remain poorly understood. Using an ARS model, the present study demonstrates that stress-induced upregulation of hepatic sEH diminishes anti-inflammatory EETs and promotes excessive IL-6 release. This peripheral inflammatory signal traverses the liver–brain axis to induce microglial activation and neuronal injury in the hypothalamus. These findings further support the potential of hepatic sEH as a therapeutic target for neurological disorders, with implications for clinical application.

## 5 Limitations of the study

While this study offers insights into sEH-driven stress-induced neuroinflammatory responses through IL-6 and the liver–brain axis, certain limitations should be recognized. In this study, we used systemic administration of the sEH inhibitor TPPU, which does not allow us to unequivocally distinguish the relative contributions of hepatic versus brain sEH to the observed neuroprotective effects. Nevertheless, the anti-neuroinflammatory effects we observed can be at least partially attributed to hepatic sEH inhibition. We acknowledge that liver-specific sEH interventions (e.g., liver-specific Ephx2 knockout or AAV-mediated liver-specific knockdown), combined with direct assessment of neurological damage, remain to be explored in order to definitively establish the causal role of hepatic sEH in this process and we have already designated this as a priority for our future studies. Additionally, although our findings establish a correlation between sEH upregulation and elevated IL-6 expression, the precise molecular mechanisms that link these two events have yet to be fully clarified. Future *in vitro* studies employing hepatocyte cell lines with sEH overexpression or knockdown would be instrumental in conclusively defining the causal signaling pathways that connect sEH activity to IL-6 transcription. In parallel, we will dissect the molecular events through which IL-6 drives neuronal injury, with the goal of fully establishing the mechanistic connection between peripheral and central events. Moreover, future studies will investigate the mechanisms linking stress-induced abnormalities in arachidonic acid (AA) metabolism and sEH-mediated IL-6 inflammatory signaling. To dissect the relative contributions of neural reflexes versus humoral mediators in stress-induced brain injury, future experiments will include vagotomy or sympathectomy to directly evaluate autonomic nerve signaling to the hypothalamus. These ongoing and planned studies will further elucidate the liver–brain axis in acute stress-induced neuroinflammation.

## Data Availability

The original contributions presented in the study are included in the article/supplementary material. Further inquiries can be directed to the corresponding authors.
